# Fungal Growth Risk Prediction and Optimal Regulation Method for Food Storage Based on the Forward Reachable Set

**DOI:** 10.3390/foods15172975

**Published:** 2026-08-25

**Authors:** Zhiyao Zhao, Mengshan Li, Yuqin Zhou, Fan Zhang, Xiaolei Sun

**Affiliations:** School of Computer and Artificial Intelligence, Beijing Technology and Business University, Beijing 100048, China; zhaozy@btbu.edu.cn (Z.Z.); shanyy378@gmail.com (M.L.);

**Keywords:** food storage, fungal growth, forward reachable set, risk prediction, optimal regulation

## Abstract

Affected by coupled environmental factors including temperature and water activity, food storage is restricted by fungal contamination, quality degradation, and energy limits. Conventional microbial growth prediction models typically rely on given initial states and environmental parameters, making it difficult to account for the effects of prior-parameter errors and thereby limiting the accurate quantification of fungal growth risk and the real-time regulation of storage environments. This paper develops a fungal growth risk prediction and optimal regulation method for food storage based on the forward reachable set (FRS). The method combines a fungal growth kinetic model for *Aspergillus flavus* with FRS theory to calculate the reachable domains of colony radius and cell states within a finite time horizon, adopts a risk margin to describe the maximum colony expansion relative to deterministic growth trajectories, and constructs a multi-objective index covering energy cost, fungal growth risk, quality loss, and control switching cost to select the optimal environmental control scheme. Numerical simulation results show that the risk margin reflects the expansion of fungal growth risk caused by the propagation and accumulation over time of prior-parameter errors, while the selected regulation strategy exhibits stronger conservatism.

## 1. Introduction

Globally, postharvest food spoilage remains a prominent problem [1,2,3]. Food generally undergoes multiple stages from postharvest handling to consumption, including sorting, packaging, transportation, storage, and sale, among which storage is an important stage connecting production and consumption. Food storage is a typical multi-physics coupled, nonlinear, time-varying dynamic system. Temperature, water activity, gas concentration and packaging conditions jointly govern microbial growth and food spoilage [4,5,6]. Fluctuating storage environments lead to widespread premature food deterioration prior to consumption, resulting in substantial food loss and public health hazards from foodborne illnesses. Previous studies identify routine warehouse inspection and real-time environmental monitoring as core fresh produce quality management measures [7,8]. Nevertheless, energy consumption creates significant economic constraints in actual storage operations. Storage environmental regulation must balance preservation performance and energy saving to optimize food safety and operating costs simultaneously [9,10,11]. In summary, food storage is a complex dynamic process affected by various environmental factors, subject to dual restrictions of food safety standards and energy costs. Systematic research into fungal growth risk prediction and optimal regulation methods is urgently required to develop safe and energy-efficient modern food storage systems.

At present, two relatively mature predictive algorithm systems have been established in predictive microbiology. The first category comprises kinetic modeling methods centered on microbial growth mechanisms. By constructing a hierarchical framework consisting of primary, secondary, and even tertiary models, these methods use experimental data obtained under specific environmental conditions to fit growth kinetic parameters coupled with environmental factors, thereby explicitly describing the quantitative relationship between microbial concentration and environmental factors. They can accurately predict microbial growth behavior under constant environmental conditions and provide interpretable and verifiable theoretical support for food safety assessment. Representative foundational studies include the predictive microbiology framework summarized by Ross and McMeekin and the dynamic microbial growth model developed by Baranyi and Roberts [12,13]. Salt concentration, pH, and storage temperature were incorporated into a Gompertz model to fit growth curves, and a predictive model was subsequently constructed using a second order polynomial to predict the growth of *Salmonella* in food [14]. The Baranyi primary model was first used to fit microbial growth at different temperatures and extract parameters such as the maximum growth rate and lag phase, after which a secondary model was used to relate these parameters to temperature, thereby enabling growth prediction under constant and varying temperature conditions [15]. Growth parameters were obtained using the Baranyi model, and the relationships between these parameters and temperature were fitted using a modified Ratkowsky model; the fitted relationships were then substituted back into the model to predict microbial growth under varying temperature conditions [16]. Although these methods exhibit strong interpretability and reliability, it remains difficult for them to achieve satisfactory prediction accuracy when applied to complex storage processes characterized by high dimensionality, nonlinearity, and strong coupling among multiple factors. The second category comprises intelligent prediction methods centered on data-driven approaches. These methods rely on machine learning models to automatically identify statistical patterns of microbial growth or food spoilage from multisource food property data and environmental sensor data without requiring explicit mechanistic assumptions. Representative data-driven studies include the use of XGBoost and the ComBase database to predict the population behavior of *Listeria monocytogenes* under multidimensional food and environmental conditions, as well as the direct prediction of *Escherichia coli* O157 growth using machine learning without separately defining primary and secondary models [17,18]. Yildirim Yalci et al. [19] developed a machine learning regression model that effectively predicted the total mesophilic bacterial count in spinach; Yucel and Tarlak [20] employed machine learning methods, including decision tree regression, to establish a microbial growth model applicable to beef; Sonwani et al. [21] proposed an intelligent prediction model based on a convolutional neural network, providing a theoretical basis for reducing food losses. Overall, data-driven methods relax the need to explicitly specify microbial growth mechanisms and are more flexible in capturing high-dimensional, nonlinear, and strongly coupled relationships among multiple environmental factors. However, their predictive performance depends strongly on the quantity, quality, representativeness, and consistency of the available data [17,22,23]. In summary, both categories of existing methods have achieved substantial progress in microbial growth modeling but retain evident limitations: mechanistic models provide relatively strong interpretability but may be constrained by predefined mathematical structures when describing microbial behavior under complex and dynamically changing environments, whereas data-driven methods can achieve high predictive accuracy but generally provide limited mechanistic interpretability [24,25]. More importantly, most existing models primarily predict microbial growth, survival, or inactivation trajectories. Although such predictions can contribute to exposure assessment within quantitative microbiological risk assessment, they do not by themselves constitute a complete microbiological risk assessment, which additionally requires hazard characterization, dose–response assessment, and risk characterization [26,27].

Food storage quality and microbial growth are significantly affected by temperature and moisture status; therefore, existing studies have mainly achieved food preservation through the regulation of storage environments. The moisture status of the storage environment is primarily described using two variables: relative humidity, which characterizes the water vapor conditions of the air surrounding the food, and water activity, which reflects the availability of water in the food matrix for microbial utilization. Regarding pretreatment before storage, a three-dimensional heat and mass transfer model was established based on the multicomponent heterogeneous structure of maize kernels and was subsequently used to optimize a multistage drying process with tempering, thereby reducing the internal temperature and moisture gradients of the kernels while shortening the drying time and providing a basis for subsequent safe storage [28]. A stepwise cooling algorithm was designed to achieve the superchilled storage of pork for low-temperature food preservation. Compared with conventional refrigeration and freezing, superchilling exhibited significant advantages in reducing drip loss and maintaining color [29]. A numerical model of temperature and humidity distributions in a potato storage environment was developed based on computational fluid dynamics. The effects of supply air temperature, velocity, and humidity on the uniformity of the storage environment were systematically analyzed, and the optimal supply air combination was identified as 3 °C, 5 m/s, and 90% RH [30]. Green peppers were used to systematically investigate the effects of different temperature and humidity combinations on respiration rate and respiratory heat. The results showed that high temperature significantly accelerated respiration, whereas high humidity effectively reduced the respiration rate [31]. To simulate the actual postharvest storage environment of wheat in regions with a Mediterranean climate, the effects of two humidity levels, 75% and 55%, on wheat quality were compared under varying temperature conditions. The results showed that, during the 180-day storage period, the moisture content of wheat increased, the test weight decreased, and the falling number increased under both humidity treatments [32]. Although the above studies clarified the effects of temperature and humidity on food deterioration during storage, they did not quantitatively characterize microbial growth dynamics under these storage conditions. Therefore, predictive modeling has been increasingly applied in combination with the intrinsic characteristics of food to characterize microbial growth responses under various environmental conditions [33]. Recently, predictive microbiology has mainly focused on the quantitative evaluation of microbial proliferation in food, providing a reliable analytical approach for preventing and controlling postharvest food spoilage [34]. A study conducted on a strawberry-based medium showed that temperature and water activity significantly affected the spore germination of *Botrytis cinerea*. The cardinal model indicated that the optimal germination temperature was 20 °C and the minimum water activity threshold was 0.858, whereas spore development could be effectively delayed when the storage temperature was below 10 °C in combination with reduced water activity [35]. Storage temperature and water activity were shown to significantly affect the growth and mycotoxin accumulation of *Fusarium* species associated with potatoes, with fungal development being substantially promoted when the temperature increased to 15 °C, and the water activity increased to 0.99 [36]. Changes in the population of *Aspergillus* species in fresh pistachios under different packaging and temperature conditions were systematically investigated. Refrigerated storage of fresh pistachios at 4 °C with suitable plastic packaging was recommended, whereas storage at room temperature for more than 8 days was not recommended [37]. Medium- and long-term temperature and humidity prediction was achieved using a smart greenhouse dataset from Weifang, Shandong Province, and the root mean square error of temperature prediction was improved, providing support for agricultural applications [38]. Most of these studies were limited to testing under specific conditions or treated the environmental variables themselves as prediction targets. However, in practical food storage, prior parameters such as the initial fungal growth state, temperature, and water activity are often difficult to determine precisely and may vary within certain ranges, making deterministic predictions based on single parameter values insufficient to fully characterize the possible fungal growth states. Moreover, fungal growth prediction and storage environment regulation are generally performed independently, making it difficult to quantify potential fungal growth risks caused by uncertainty and incorporate them into regulation decisions.

To integrate the fungal growth prediction mechanism, incorporate fungal growth risk into the objectives of dynamic environmental regulation, and meet the integrated requirements for fungal growth risk prediction and optimal regulation during food storage, this study proposes a fungal growth risk prediction and optimal regulation method for food storage based on the forward reachable set (FRS). In this study, fungal growth risk is defined as the potential growth expansion caused by prior-parameter errors and is quantified by the difference between the maximum reachable colony radius under uncertainty and the corresponding deterministic prediction. Here, this risk characterizes the additional fungal growth potential beyond the deterministic prediction, indicating the extent to which the deterministic trajectory may underestimate possible colony expansion. The proposed method aims to achieve the quantification of fungal growth risk and the coordinated optimal regulation of the storage environment, thereby providing a theoretical basis and technical support for ensuring food storage safety, improving preservation effectiveness, and reducing energy consumption. The main contributions of this study are as follows:

1. First, the fungal growth kinetic model is integrated with the forward reachable set to predict fungal growth over a finite prediction horizon while considering errors in the prior parameters.
2. Second, a time-varying risk margin indicator is designed to construct a fungal growth risk prediction method.
3. Finally, based on the fungal growth risk prediction results, a regulation algorithm for the food storage process is developed by comprehensively considering fungal growth risk and energy consumption indicators.

The remainder of this article is organized as follows: Section 1 presents the introduction and reviews microbial growth prediction and food storage environment regulation algorithms; Section 2 introduces the proposed algorithm; Section 3 evaluates the numerical performance of the proposed algorithm through numerical simulations; and Section 4 presents the conclusions.

## 2. Materials and Methods

This section presents the fungal growth model, the fungal growth risk prediction algorithm based on the FRS, and the optimal regulation method for storage strategies. For clarity, the main mathematical symbols used in the proposed method are summarized in Table 1.

### 2.1. Fungal Growth Model

The classical Baranyi–Roberts model was widely used for microbial growth prediction because it could simultaneously describe microbial growth and changes in the physiological state of the cells [13,39]. This model also provided a basic framework for characterizing the relationship between environmental factors and the microbial growth process. However, because it only provided a general term for the maximum specific growth rate, the coupled effects of key environmental factors, including temperature, water activity, and pH, required further quantification. Therefore, subsequent studies introduced temperature and water activity response mechanisms into the Baranyi–Roberts model. Considering the growth characteristics of *Aspergillus flavus* on pistachios, colony radius was selected as the growth indicator, and a coupled relationship among temperature, water activity, and the maximum radial growth rate was established, in which the maximum radial growth rate was expressed as the product of the optimal radial growth rate, a temperature response subfunction, and a water activity response subfunction [40]. In summary, this model was supported by both clearly defined nonlinear dynamic characteristics and an environmental coupling mechanism. Therefore, this study adopted the model as the basic framework to provide theoretical and model support for subsequent fungal growth risk prediction and optimal regulation.

Following the radial growth formulation established in the above studies [40], this study retained colony radius as the growth state variable. By jointly taking the colony radius y(t) and the physiological state of the cells q(t) as the system state vector $x\left(t\right)={\left[y\left(t\right),q\left(t\right)\right]}^{T}$, which was hereafter denoted as $x={\left[y,q\right]}^{T}$ for simplicity, a two-dimensional dynamic system was constructed:

(1) $$\left[\begin{array}{c} \dot{y}\\ \dot{q} \end{array}\right]=\left[\begin{array}{c} \mu_{R}\left(T,a_{w}\right)\cdot \frac{q}{1+q}\left(1-e^{y-y_{M}}\right)\\ \mu_{R}\left(T,a_{w}\right)\cdot q \end{array}\right]$$

where *t* denoted time, $y_{M}$ denoted the upper limit of the fungal colony radius, $\mu_{R}\left(T,a_{w}\right)$ denoted the maximum radial growth rate, *T* denoted temperature, and $a_{w}$ denoted water activity. In addition,

$$\begin{array}{c} \mu_{R}\left(T,a_{w}\right)=\mu_{opt}\cdot \tau \left(T\right)\cdot \rho \left(a_{w}\right)\\ \tau \left(T\right)=\frac{{\left(T-T_{min}\right)}^{2}\cdot \left(T-T_{max}\right)}{\left(T_{opt}-T_{min}\right)\cdot \left[\left(T_{opt}-T_{min}\right)\left(T-T_{opt}\right)-\left(T_{opt}-T_{max}\right)\left(T_{opt}+T_{min}-2T\right)\right]}\\ \rho \left(a_{w}\right)=\frac{{\left(a_{w}-a_{w_{min}}\right)}^{2}\cdot \left(a_{w}-1\right)}{\left(a_{w_{opt}}-a_{w_{min}}\right)\cdot \left[\left(a_{w_{opt}}-a_{w_{min}}\right)\left(a_{w}-a_{w_{opt}}\right)-\left(a_{w_{opt}}-1\right)\left(a_{w_{opt}}+a_{w_{min}}-2a_{w}\right)\right]} \end{array}$$

where $\mu_{opt}$ denoted the optimal radial growth rate, τ(T) denoted the temperature response subfunction, $\rho (a_{w})$ denoted the water activity response subfunction, $T_{min}$ denoted the minimum growth temperature, $T_{max}$ denoted the maximum growth temperature, $T_{opt}$ denoted the optimal growth temperature, $a_{w_{min}}$ denoted the minimum water activity, and $a_{w_{opt}}$ denoted the optimal water activity.

### 2.2. Fungal Growth Risk Prediction Algorithm Based on the FRS

Traditional predictive microbiology models generally predicted colony growth using fixed initial states and environmental parameters. Under ideal experimental conditions, these predictive models could adequately describe microbial growth. However, during actual food storage, the initial fungal growth state was often difficult to measure with high accuracy. In particular, when the initial colony size was small or the fungi were in the early growth stage, the true initial state was generally more appropriately represented in interval form. Meanwhile, environmental factors such as temperature and water activity were inevitably subject to disturbances during transportation and storage. These uncertainties continuously accumulated throughout fungal growth, thereby indirectly causing deviations between the prediction results and the actual fungal growth state and making it difficult for traditional predictive models to comprehensively reflect the potential evolution range of colony growth. The Lax–Friedrichs method could directly characterize the evolution of state sets under system dynamics constraints, thereby providing rigorous safety boundary analysis for dynamic systems with uncertainty. Reachability set theory was used to characterize the set of all states that a dynamical system could reach within a given time horizon and could be classified into the FRS and the backward reachable set (BRS) according to the direction of temporal evolution [41,42]. The FRS started from an initial state set and characterized the complete set of states that the system might reach in the future under given dynamics constraints, control inputs, and disturbances, whereas the BRS took a target state set as the terminal set and described all initial states from which the system could reach the target set within a finite time horizon. This theory has been widely applied to safety verification of autonomous vehicles, stability analysis of power systems, robot motion planning, verification of neural network control systems, and safety assessment of systems subject to stochastic disturbances [43,44,45,46]. Considering the advantages of reachability sets, this study aimed to develop a fungal growth risk prediction algorithm based on the FRS to characterize the evolution range and risk level of fungal growth in the presence of prior-parameter errors.

The fungal growth model in Equation (1) was essentially a continuous-time nonlinear dynamical system and could be expressed as

(2) $$\dot{x}=f\left(x,t,u\right),$$

where $u={\left[T,a_{w}\right]}^{T}$. Since the initial state was difficult to quantify accurately, this paper set its value range to mitigate the influence of parameter errors on prediction accuracy. The initial state set was defined as

(3) $$X_{0}=\left\{\left(y_{0},q_{0}\right)|\underline{y_{0}}\leq y_{0}\leq \bar{y_{0}},\;\underline{q_{0}}\leq q_{0}\leq \bar{q_{0}}\right\}.$$

Here, $\underline{y_{0}}$ and $\bar{y_{0}}$ denoted the lower and upper bounds of the initial colony radius, respectively, while $\underline{q_{0}}$ and $\bar{q_{0}}$ denoted the lower and upper bounds of the initial physiological state, respectively. Similarly, the temperature and water-activity were also defined with corresponding value intervals rather than fixed single values. The control input set was defined as

(4) $$U=\left\{\left(T,a_{w}\right)|\underline{T}\leq T\leq \bar{T},\;\underline{a_{w}}\leq a_{w}\leq \bar{a_{w}}\right\}.$$

Here, $\underline{T}$ and $\bar{T}$ denoted the lower and upper bounds of temperature, respectively, while $\underline{a_{w}}$ and $\bar{a_{w}}$ denoted the lower and upper bounds of water activity, respectively.

The FRS started from the initial state set and characterized all states that the system might reach in the future under the system dynamics and the control input set. The fungal growth model described above had a deterministic continuous-time dynamic structure, while both the initial state and environmental inputs were subject to bounded uncertainty, thereby satisfying the basic assumptions for applying forward reachability analysis. Therefore, this method could be used to characterize the propagation of prior-parameter errors associated with the initial fungal growth state, temperature, and water activity through the growth dynamics and determine the upper bound that the colony radius might reach. Specifically, the level set method was first used to represent $X_{0}$ as the zero sublevel set of the scalar function $\phi_{0}\left(x\right)$ defined over the state space:

$$\phi_{0}\left(x\right)=\left\{\begin{array}{c} {\underline{y}}_{0}-y_{0},\\ y_{0}-{\bar{y}}_{0},\\ {\underline{q}}_{0}-q_{0},\\ q_{0}-{\bar{q}}_{0}, \end{array}\right.$$

$$X_{0}=\left\{x\in R^{2}|\phi_{0}\left(x\right)⩽0\right\}.$$

At this point, the FRS of fungal growth originating from the initial state set $X_{0}$ under the vector field *f* could be obtained by solving for the viscosity solution [47,48] of the corresponding Hamilton–Jacobi equation [49].

Based on model (2), the Hamilton–Jacobi equation for the fungal growth model during food storage was expressed as

(5) $$\begin{array}{cc} \frac{\partial \phi (x,t)}{\partial t}+H\left(x,p\right) & =0,\;x\in R^{2},\;t⩾0,\\ \phi (x,t) & =\phi_{0}\left(x\right),\;x\in R^{2},\;t=0, \end{array}$$

where the Hamiltonian function H(x,p) is given by

$$H\left(x,p\right)=\max_{u\in U}p^{T}f\left(x,t,u\right),$$

where $p=\frac{\partial \phi (x,t)}{\partial x}$ denoted the Hamiltonian costate vector. By solving the above equations, the FRS at time *t* was denoted by $X_{t}$ and expressed as

(6) $$X_{t}=\left\{x\in R^{2}|\phi \left(x,t\right)\leq 0\right\}.$$

Following [50,51], the FRS of the fungal growth state was numerically computed by considering the dynamic characteristics of the fungal growth model for food storage. First, the Hamiltonian function was expanded as

$$H\left(x,p\right)=\max_{T\in \left[\underline{T},\bar{T}\right],\;a_{w}\in \left[\underline{a_{w}},\bar{a_{w}}\right]}\left(\frac{\partial \phi (x,t)}{\partial y}\cdot \frac{q}{1+q}\left(1-e^{\left(y-y_{M}\right)}\right)+\frac{\partial \phi (x,t)}{\partial q}\cdot q\right)\mu_{R}\left(T,a_{w}\right).$$

For computational simplification, let

$$G=\frac{\partial \phi (x,t)}{\partial y}\cdot \frac{q}{1+q}\left(1-e^{\left(y-y_{M}\right)}\right)+\frac{\partial \phi (x,t)}{\partial q}\cdot q.$$

Considering the characteristics of the environmental response function $\mu_{R}\left(T,a_{w}\right)$, H(x,p) could be decomposed into two cases as follows:

$$H\left(x,p\right)=\left\{\begin{array}{cc} \mu_{opt}\cdot \tau {\left(T\right)}_{\max}\cdot \rho {\left(a_{w}\right)}_{\max}\cdot G, & G⩾\;0\\ \mu_{opt}\cdot \tau {\left(T\right)}_{\min}\cdot \rho {\left(a_{w}\right)}_{\min}\cdot G, & G<0 \end{array}\right.,$$

where $\tau {\left(T\right)}_{\max}$ denoted the maximum value of τ(T) over the temperature control input interval, and $\tau {\left(T\right)}_{\min}$ was defined analogously; $\rho {\left(a_{w}\right)}_{\max}$ denoted the maximum value of $\rho (a_{w})$ over the water activity control input interval, and $\rho {\left(a_{w}\right)}_{\min}$ was defined analogously.

Subsequently, at the spatial discretization level, the Lax–Friedrichs scheme was used to approximate the Hamiltonian function H(x,p) to ensure the monotonicity and stability of the numerical scheme. The state space was discretized using the grid *g*. For the adjacent grid points $x_{j}$, the left derivative $p^{-}$ and right derivative $p^{+}$ could be expressed as follows:

$$p^{-}=D_{x}^{-,ENO2}\phi \left(x_{j},t\right),p^{+}=D_{x}^{+,ENO2}\phi \left(x_{j},t\right),$$

where $D_{x}^{-,ENO2}$ and $D_{x}^{+,ENO2}$ denote the left and right second-order essentially nonoscillatory (ENO) upwind difference operators in the state space, respectively [52]. The derivative approximations were computed componentwise along the state vector. Averaging the left and right derivatives yielded the central gradient approximation:

$$p=\frac{p^{-}+p^{+}}{2}.$$

Moreover, to ensure the numerical stability of the Lax–Friedrichs scheme, suppress oscillations during discretization, and maintain the smoothness and computational stability of the evolving reachable set boundary, a dissipation coefficient $k_{i}$ was introduced in the *i*-th dimension (i=1,2), whose value was taken as the upper bound of the absolute partial derivative of the Hamiltonian function with respect to the corresponding gradient component, $k_{i}=\max_{p_{i}}\left|\frac{\partial H}{\partial p_{i}}\right|$. Combined with *G*, the dissipation coefficients were calculated as follows:

$$k_{1}=\mu_{opt}\cdot \tau {\left(T\right)}_{\max}\cdot \rho {\left(a_{w}\right)}_{\max}\left|\frac{q}{1+q}\left(1-e^{\left(y-y_{M}\right)}\right)\right|,$$

$$k_{2}=\mu_{opt}\cdot \tau {\left(T\right)}_{\max}\cdot \rho {\left(a_{w}\right)}_{\max}\left|q\right|.$$

Finally, the Lax–Friedrichs approximation of H(x,p) was given by:

$$H_{LF}=H\left(x,\frac{p^{+}+p^{-}}{2}\right)-\frac{1}{2}\sum_{i=1}^{2}k_{i}\left(p_{i}^{+}-p_{i}^{-}\right).$$

Further, an explicit integrator satisfying the CFL stability condition was applied in the temporal direction to iteratively obtain the function ϕ(x,t+Δt), with the basic form given as follows:

$$\phi \left(x,t+\Delta t\right)=\phi \left(x,t\right)+\Delta t\frac{\partial \phi (x,t)}{\partial t}.$$

The FRS at time τ was then obtained. By controlling the time step Δt, the numerical results could approximate the continuous-time evolution process.

Finally, based on the dynamic evolution results of the forward reachable set, a time-dependent risk margin vector δ(t) was constructed to perform fungal growth risk prediction and quantify the potential expansion of fungal growth in the system state space. Specifically, let the number of prediction steps be $N_{p}$ and the regulation period be Δt; then the prediction time points were $t=n\Delta t\;(n=1,2,\ldots ,N_{p})$. At each prediction time point *t*, the degree of expansion of the farthest point $y_{\max}\left(t\right)$ on the reachable set boundary relative to the deterministic prediction trajectory $\hat{y}\left(t\right)$ of the system under the corresponding temperature and water activity values was calculated as follows:

(7) $$\delta \left(t\right)=y_{\max}\left(t\right)-\hat{y}\left(t\right).$$

It should be emphasized that introducing this risk margin did not imply that the system would inevitably encounter the worst-case extreme condition. Its primary purpose was to objectively characterize the divergence and expansion trends that the system states might exhibit under uncertain conditions. Therefore, δ(t) essentially served as forward-looking risk awareness information, aiming to effectively compensate for the inherent limitation of traditional predictive models in providing overly optimistic estimates of future fungal growth risk. In addition, as the prediction horizon increased, the cumulative effects of system uncertainty gradually intensified, causing the risk margin to generally exhibit a dynamic trend of continuous expansion over time.

### 2.3. Optimal Regulation of Storage Strategies Under Fungal Growth Risk Prediction

In practical storage systems, equipment was constrained by physical inertia and operating costs, making it difficult to achieve high frequency continuous adjustment of environmental inputs, while maintaining low temperature and water activity levels over prolonged periods significantly increased system energy consumption. Meanwhile, the optimal regulation process based on fungal growth risk prediction during food storage was a nonlinear dynamic optimization problem, and directly solving it in the continuous environmental input space would substantially increase computational complexity. To balance engineering feasibility and real time optimization efficiency, this study discretized the environmental inputs and transformed the optimization problem into a decision making problem over a finite set of regulation strategies. Based on these considerations, a discrete regulation mode library comprising representative steady state combinations of temperature and water activity was constructed:

$$M=\{m_{1},m_{2},\ldots ,m_{N}\}.$$

Here, the *i*-th regulation mode was $m_{i}={\left[T_{i},a_{w,i}\right]}^{T}$, where $T_{i}$ denoted the target storage temperature under this mode and $a_{w,i}$ represented the corresponding target water activity level for this mode. And *N* denotes the number of modes in the mode library. Clearly, the discrete regulation mode library for temperature and water activity could not encompass all possible environmental states, but it could simulate representative regulation paths encountered during actual storage regulation. In addition, the regulation strategy was constrained to a piecewise constant signal comprising $N_{p}$ segments, and the input at each prediction time point *t* had to be selected from the mode library *M*. Thus, any candidate strategy $\pi =[m\left(\Delta t\right),m\left(2\Delta t\right),\ldots ,m\left(N_{p}\Delta t\right)]$ was formed by freely combining the discrete regulation modes.

To balance multiple factors, including energy consumption, fungal growth risk, quality, and regulation smoothness, during food storage, this study constructed the following comprehensive performance evaluation index:

(8) $$J=\sum_{t=\Delta t}^{N_{p}\Delta t}\left(\alpha E(t)+\beta R(t)+\eta Q(t)\right)+\sum_{t=\Delta t}^{(N_{p}-1)\Delta t}\lambda S\left(t\right).$$

Here, E(t), R(t), Q(t), and S(t) denoted the energy consumption cost, fungal growth risk cost, quality deterioration cost, and regulation switching cost at time point *t*, respectively. The parameters α, β, η, and λ were the weighting coefficients of the corresponding cost terms and were used to balance the comprehensive optimization objectives of system energy consumption, fungal growth risk, quality preservation, and regulation smoothness.

First, because lower-temperature environments generally entailed greater refrigeration expenditure, the energy consumption term E(t) was used to quantitatively describe the refrigeration cost induced by temperature regulation. In this study, the quadratic deviation between the current regulation temperature and the preset ambient temperature was used to characterize the overall energy consumption level:

$$E\left(t\right)={\left(T\left(t\right)-T_{env}\left(t\right)\right)}^{2},$$

where T(t) denoted the current regulation temperature of the system, and $T_{env}\left(t\right)$ denoted the preset ambient temperature. This indicator reflected the relative refrigeration intensity required to maintain the target storage temperature under different ambient conditions: the greater the temperature difference, the higher the refrigeration load borne by the system and the greater the corresponding energy consumption cost. Incorporating this term into the comprehensive performance index prevented the optimizer from suppressing fungal growth solely by continuously reducing the temperature, thereby achieving a reasonable balance between fungal growth control and refrigeration energy consumption.

Second, the risk term R(t) was specifically used to quantify the severity of fungal growth risk when the fungal colony radius *y* approached or exceeded the growth warning boundary, and was defined as the following piecewise function:

$$R\left(t\right)=\left\{\begin{array}{cc} 0, & y\left(t\right)⩽y_{\mathrm{safe}}\\ 50{\left(y\left(t\right)-y_{\mathrm{safe}}\right)}^{2}, & y_{\mathrm{safe}}<y\left(t\right)⩽y_{\mathrm{safe}}+0.1\\ e^{c_{\mathrm{Risk}}\cdot \max\left(0,y\left(t\right)-y_{\mathrm{safe}}\right)}-1, & y\left(t\right)>y_{\mathrm{safe}}+0.1 \end{array}\right.,$$

where $y_{\mathrm{safe}}$ denoted the specified fungal growth warning boundary, and $c_{\mathrm{Risk}}$ denoted the risk exponential growth coefficient. This risk function adopted a piecewise mechanism with progressively increasing penalties: when the risk state had just exceeded the growth warning boundary, a quadratic term was used to provide continuous and smooth risk penalization, thereby avoiding severe system fluctuations caused by abrupt changes in the risk function during optimization; when the risk increased further, an exponential growth form was used to rapidly increase the risk cost, thereby enhancing the optimizer’s sensitivity to serious threshold exceedance risks and causing the system to exhibit a more conservative regulation tendency in high-risk regions.

Third, the quality deterioration cost Q(t) was used to quantify the extent to which the actual regulation conditions deviated from the predefined quality reference conditions. In this study, the quality reference conditions referred to combinations of temperature and water activity that were preset for a specific food and storage stage to maintain quality attributes such as color, texture, moisture, and flavor. These reference conditions did not represent a universally optimal environment applicable to all foods; rather, they were jointly determined by the food type and quality preservation requirements, and their specific values could be set according to the actual circumstances. The deviations of temperature and water activity from their respective reference conditions were integrated as follows:

$$Q\left(t\right)={\left(T\left(t\right)-T_{q}\left(t\right)\right)}^{2}+400{\left(a_{w}\left(t\right)-a_{w,q}\left(t\right)\right)}^{2},$$

where $T_{q}\left(t\right)$ and $a_{w,q}\left(t\right)$ denotes the reference temperature and reference water activity for maintaining food quality, respectively. Given the difference in magnitude between temperature *T* and water activity $a_{w}$, the weight assigned to the water activity deviation term was appropriately increased to balance the effects of the two variables.

Finally, the regulation switching cost term S(t) was used to suppress abrupt changes in the regulation strategy between adjacent prediction time points, thereby ensuring the smoothness and engineering feasibility of the regulation strategy. It was mathematically defined as follows:

$$S\left(t\right)={\left(T(t+\Delta t)-T(t)\right)}^{2}+{\left(a_{w}\left(t+\Delta t\right)-a_{w}\left(t\right)\right)}^{2}.$$

This term could effectively prevent excessively frequent adjustments of temperature and water activity, thereby reducing the implementation burden and equipment wear in actual storage systems.

For any of the candidate temperature–water activity regulation strategies described above, given the initial fungal growth state and model parameters, temperature and water activity were treated as constants within each regulation period. The corresponding maximum radial growth rate $\mu_{R}\left(T,a_{w}\right)$ was then calculated, and the fungal growth dynamics equations over that period were numerically integrated using the explicit Runge–Kutta method [53]. Subsequently, the terminal state of the current period was used as the initial state of the next period for sequential propagation, ultimately yielding a colony radius prediction sequence y(t) uniquely corresponding to the candidate regulation strategy. Clearly, this prediction process did not account for the propagation of environmental disturbances or initial state uncertainty. Drawing on the definition of the nominal prediction trajectory in the field of control [54], this study defined the baseline prediction result directly obtained from the fungal growth model under a given initial state, model parameters, and temperature–water activity strategy as the nominal fungal growth prediction trajectory. Therefore, this study further incorporated the risk margin δ(t) to obtain the fungal growth risk prediction result $\tilde{y}\left(t\right)$ for the food storage process. The risk prediction result was calculated as follows:

(9) $$\tilde{y}\left(t\right)=y\left(t\right)+\delta \left(t\right).$$

In the subsequent search for the optimal regulation strategy, all candidate regulation strategies were evaluated. The temperature–water activity strategies that minimized the comprehensive performance index under the two prediction methods were determined separately, and their regulation modes, fungal growth trajectories, and risk control effects were further compared to analyze the influence of introducing the risk margin on the optimal regulation decision.

### 2.4. Applicability and Limitations of the Proposed Method

This study proposed a fungal growth risk prediction and optimal regulation method for food storage based on the FRS to address the limitation that traditional predictive microbiology models could only predict a single growth trajectory and could not adequately reflect the effects of initial state errors and environmental fluctuations. The proposed method consisted of two parts: risk prediction and optimal regulation. The former used the FRS to obtain the range of fungal growth states that might be reached and employed the risk margin to characterize its expansion relative to the nominal prediction trajectory. The latter comprehensively considered energy consumption, fungal growth, food quality deterioration, and regulation switching costs to select a storage strategy that balanced multiple objectives. In addition, the proposed framework could potentially be extended to other classes of microorganisms by replacing the current fungal growth model with microorganism-specific kinetic models and corresponding state variables.

Despite these advantages, the proposed method still had certain limitations. First, the optimization was performed only within a predefined finite regulation mode library. Therefore, the resulting strategy was the optimal solution among the candidate modes rather than the global optimum in the continuous environmental input space, and an inappropriate mode library might reduce the regulation accuracy. Second, the water activity parameter in the current fungal growth model may be difficult to regulate directly in practical scenarios, while colony radius may not be an appropriate state variable for other classes of microorganisms. Therefore, the corresponding kinetic model, state variables, and control variables should be adjusted according to microorganism-specific growth characteristics and practical storage conditions. Future studies will further investigate the applicability of the proposed framework to other classes of microorganisms using appropriate growth models and state variables. Third, this study was mainly based on numerical simulations and did not statistically validate the prediction results using data from actual food storage experiments. Future studies should combine experimental observations with appropriate statistical evaluation metrics to further assess the prediction accuracy and practical applicability of the proposed method.

## 3. Results and Discussion

This section presents the numerical simulation results of the proposed algorithm. In the fungal growth model containing a logistic inhibition term, the upper limit of the colony radius, $y_{M}$, essentially represented the effective carrying boundary imposed by limited nutrient availability and spatial constraints. When $y_{M}$ was set too low, the logistic inhibition effect dominated the system dynamics prematurely, leading to an unreasonable colony growth pattern and causing the colony to enter the saturation plateau too early. Conversely, when $y_{M}$ was set far above the colony size attainable within the study period, the logistic inhibition term became approximately inactive within the prediction horizon, and the model behavior approached linear radial growth. Based on these considerations and with reference to [40], the relevant growth parameters of *A. flavus* on pistachios were listed in Table 2.

### 3.1. Numerical Simulation Setup

The proposed algorithm was implemented in MATLAB R2012b on a Windows 11 operating system. The numerical computation of the forward reachable set was carried out using the Toolbox of Level Set Methods, Version 1.1. In the numerical scheme, the Hamiltonian term was approximated using the Lax–Friedrichs numerical flux, and the spatial derivatives were discretized by a second-order essentially nonoscillatory upwind scheme. The time integration was performed using a second-order time integrator constrained by the Courant–Friedrichs–Lewy condition. The numerical stability of the Hamilton–Jacobi solver was mainly controlled by the CFL condition. In this study, the CFL factor was set to 0.75. In addition, the Lax–Friedrichs numerical flux and global Lax–Friedrichs artificial dissipation were used to enhance the stability of the reachable set propagation.

### 3.2. Numerical Simulation 1: Analysis of Fungal Growth Risk Prediction Results Based on the FRS

Previous studies have identified temperature and water activity as key factors affecting the growth of *A. flavus* and aflatoxin production in pistachios. Relevant studies covered temperatures of 10–42 °C and water activity of 0.85 or higher [40,55]. Accordingly, 18 °C and 0.83 were selected as the baseline temperature and water activity, respectively, to represent mild storage conditions with relatively slow fungal growth. For the initial state, $y_{0}=0.12$ was set to represent a small initial colony relative to $y_{M}=10$, while $q_{0}=0.06$ was adopted to represent low initial physiological activity according to the physiological interpretation of q(t) in the Baranyi–Roberts model [13]. Since the true values of prior parameters including the initial fungal growth state, temperature, and water activity could not be determined in advance, a single preset value would lead to non-negligible errors in the prior parameters. Numerical Simulation 1 therefore took the above specified initial states $y_{0}$ and $q_{0}$, together with the corresponding growth rate $\mu_{R}$, as the central values and described the environmental inputs and initial fungal growth states using intervals. Specifically, temperature and water activity were restricted to [10 °C, 20 °C] and [0.75,0.85], respectively, while the ranges of the initial states $y_{0}$ and $q_{0}$ were extended to [0.08,0.15] and [0.0425,0.08], respectively. The storage time *t* was set to 8 days, with each day taken as a prediction time point, i.e., Δt=1 and $N_{p}=8$.

**Figure 1:**
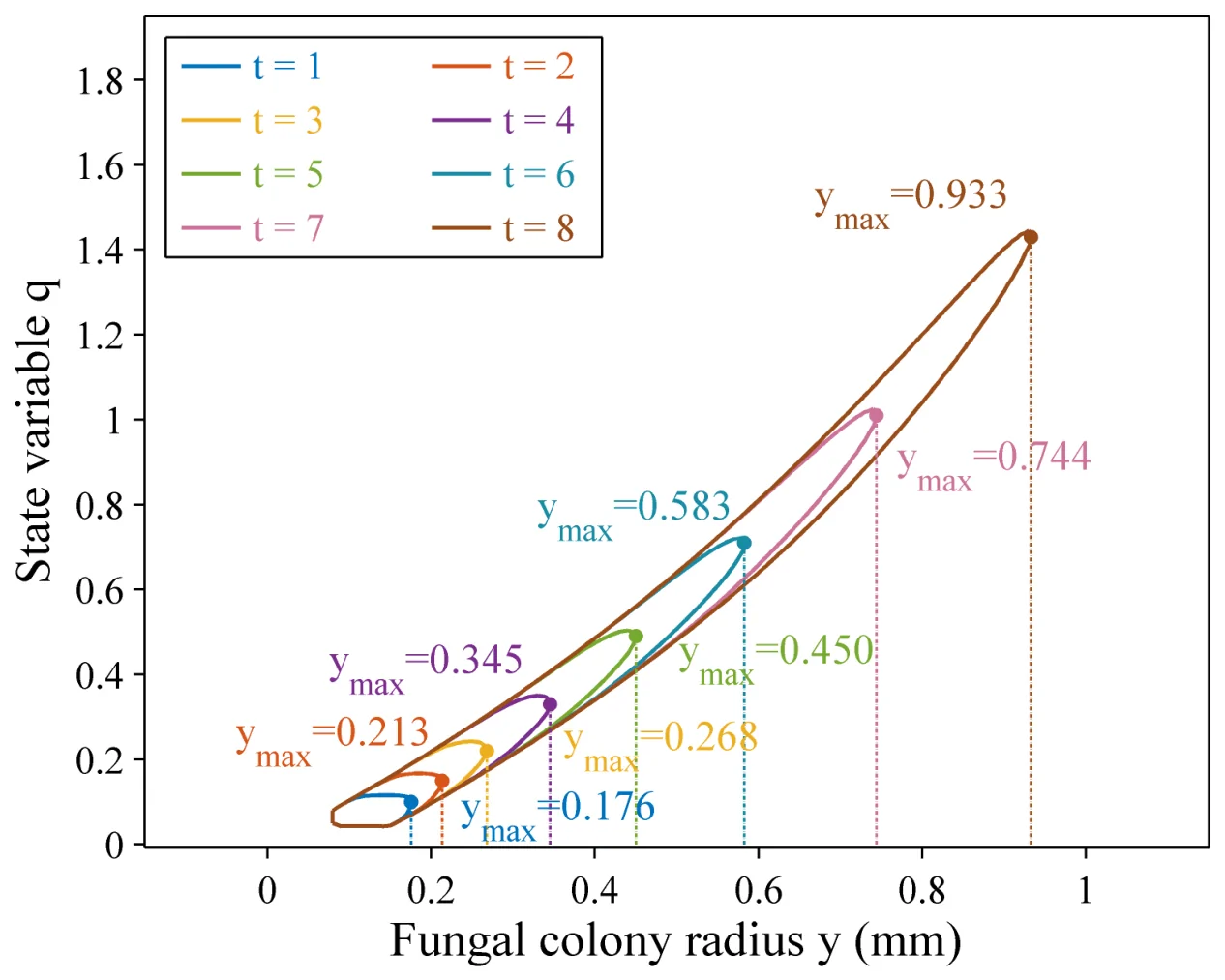
Prediction results for fungal colony radius based on the FRS.

Fungal growth prediction during food storage based on the FRS could simultaneously account for the range of states that the system might reach under all admissible initial states and environmental input conditions, thereby obtaining the overall evolution boundary of the fungal growth state at future time points. At each prediction time point, this study extracted the farthest reachable point $y_{\max}$ along the colony radius dimension from the boundary of the reachable set and regarded it as the most adverse value that *y* might attain under the current uncertain conditions; the calculation results were shown in Figure 1. The analysis indicated that, on the one hand, during the early prediction stage, the FRS was mainly concentrated in regions characterized by smaller colony radii and lower values of the physiological state of the cells, indicating that the fungi remained in the adaptation stage to the storage environment, with limited physiological activity and colony expansion. Consequently, their immediate impact on the food was relatively small, which also provided a time window for environmental regulation. As storage time increased, the reachable set continued to expand along the dimensions of colony radius and the physiological state of the cells, indicating that the fungi gradually completed environmental adaptation, exhibited enhanced physiological activity, and promoted an increase in colony radius. At this stage, some admissible fungal growth trajectories might enter the rapid growth stage earlier, causing the colony expansion outcomes to gradually diverge and broadening the range of possible colony-radius outcomes. On the other hand, the farthest reachable value $y_{\max}$ along the colony radius dimension grew relatively slowly during the early prediction stage but increased rapidly from 0.450 on day 5 to 0.933 on day 8. The maximum radius that the colony might reach under uncertainty increased rapidly during the middle and late stages of storage, accompanied by a widening range of possible colony-radius outcomes and a marked increase in the risks of mold spoilage and quality deterioration. Without timely regulation of temperature and water activity, the acceptable storage period of the food might be further shortened.

In summary, the evolution characteristics of the physiological state of the cells and colony radius could be jointly used to identify the critical period during which fungi transitioned from the adaptation stage to the rapid growth stage, thereby providing a basis for the timely regulation of the storage environment.

**Figure 2:**
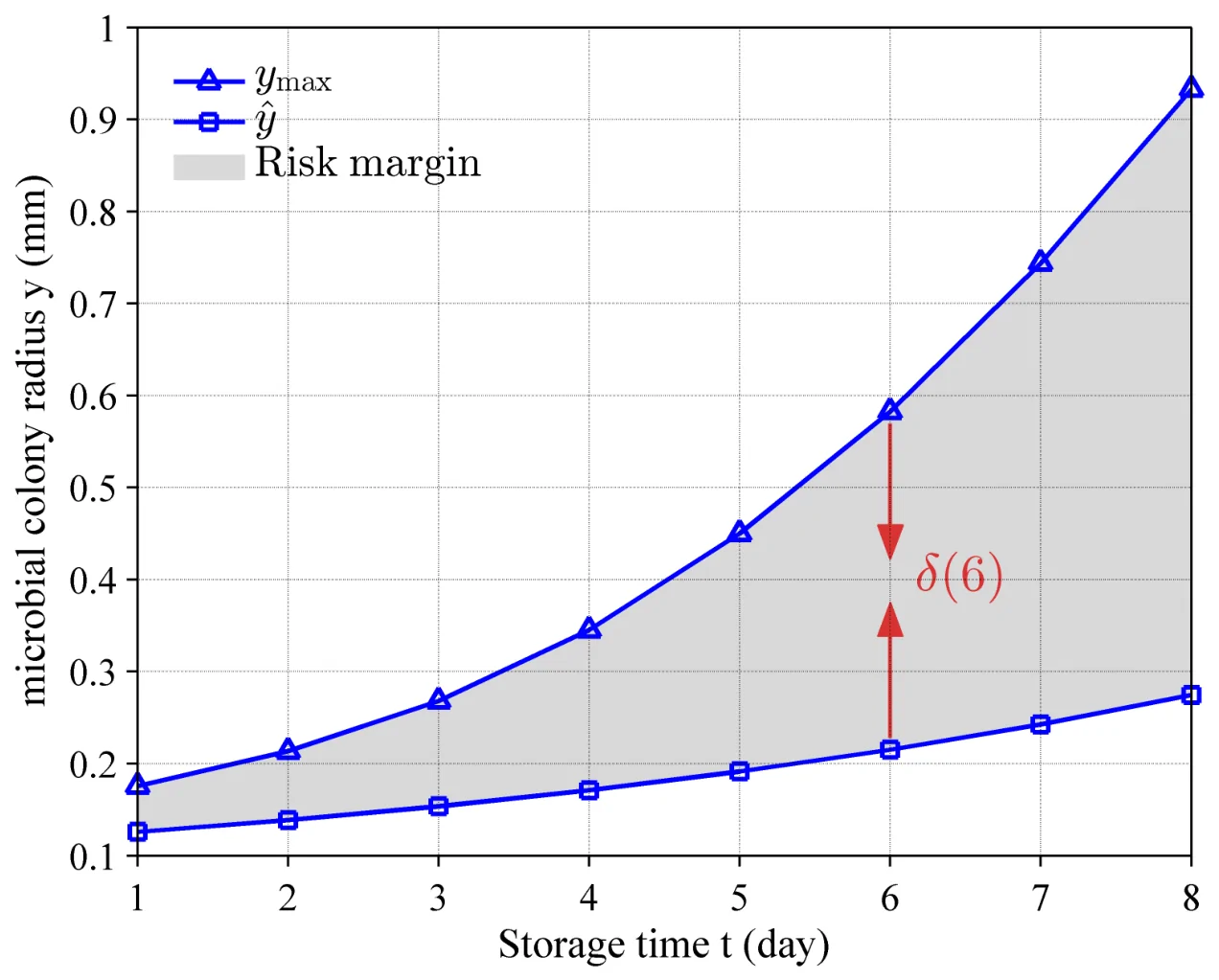
Evolution of the risk margin based on the FRS.

The fungal growth risk prediction results were shown in Figure 2. As shown in Figure 2, as the prediction time increased, the shaded region enclosed by the nominal prediction trajectory $\hat{y}\left(t\right)$ and the farthest reachable point $y_{\max}\left(t\right)$ continued to expand. This indicated that even when the nominal prediction trajectory remained relatively stable overall, the state expansion induced by uncertainty became increasingly pronounced over time. In other words, traditional prediction methods might underestimate the potential future fungal growth risk, whereas the risk margin could further reveal the propagation and accumulation trends of fungal growth risk overlooked by the nominal prediction. Therefore, this study ultimately constructed a risk margin vector that varied with the prediction time and incorporated it into the subsequent finite-time optimal regulation method to enhance the system’s ability to account for the propagation of potential fungal growth risk.

### 3.3. Numerical Simulation 2: Comparison of Optimal Regulation Results for Storage Strategies Under Nominal Prediction and Fungal Growth Risk Prediction

**Figure 3:**
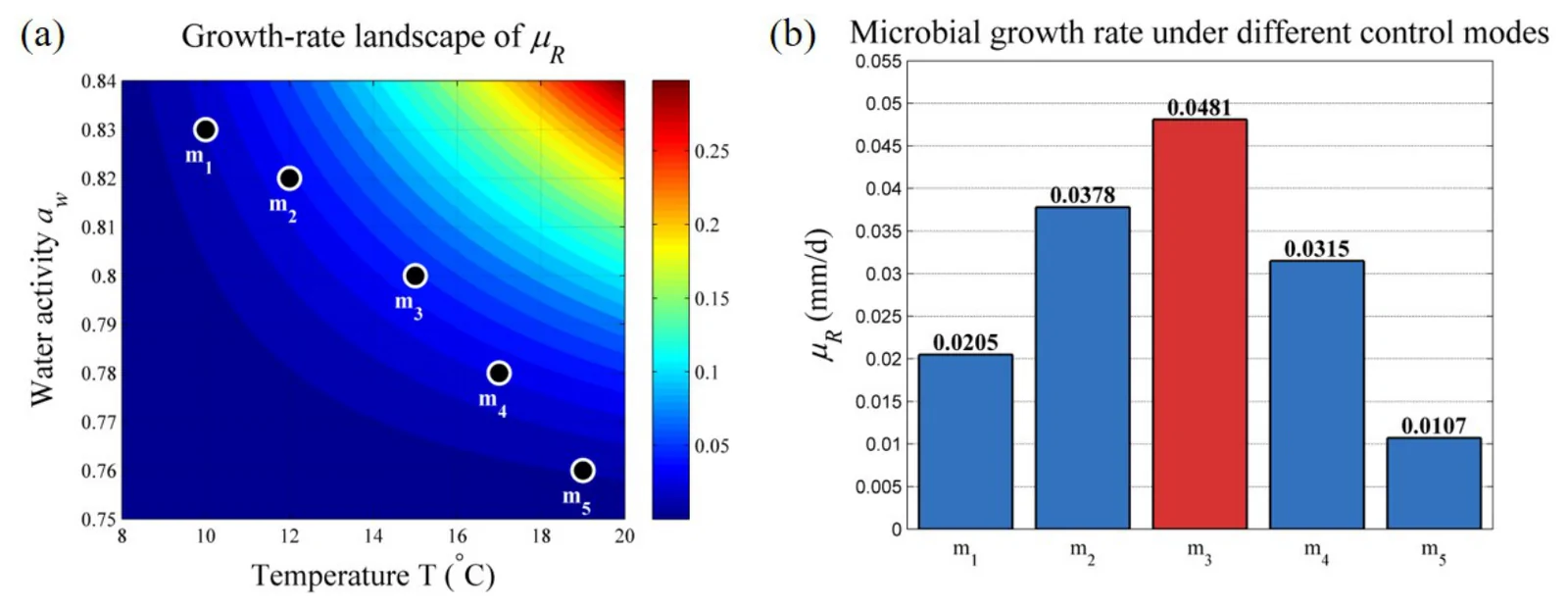
Contour plot of the maximum radial growth rate under the coupled effects of temperature and water activity (**a**) and comparison of the maximum radial growth rate under discrete regulation modes (**b**).

Considering the different roles of temperature and water activity in fungal growth control and storage cost, the regulation mode library was designed by assigning each mode a specific temperature level and a corresponding water activity level. Lower-temperature modes provided stronger fungal growth inhibition but required higher cooling costs, whereas higher-temperature modes reduced the cooling burden and therefore required stricter water activity control to limit fungal growth. Based on this consideration, a finite set of candidate regulation modes was constructed for subsequent strategy optimization, as shown in Table 3, and the corresponding calculated maximum radial growth rates were shown in Figure 3.

In the storage cost function, the reference ambient temperature was set as $T_{env}=\left[18,18,18,17,17,16,16,15\right]$. Meanwhile, to account for food quality preservation requirements, the reference temperature sequence for quality preservation was set as $T_{q}=\left[15,15,15,14,13,12,11,10\right]$, and the reference water activity sequence for quality preservation was set as $a_{w,q}=\left[0.80,0.80,0.80,0.81,0.81,0.82,0.83,0.83\right]$. Furthermore, α=1 was used as the reference weight because the energy consumption term E(t) was already expressed as a squared temperature deviation and therefore exhibited a relatively large numerical scale under low-temperature regulation conditions. The fungal growth risk term R(t) was a threshold-dependent penalty that remained zero below the growth warning boundary and increased rapidly only when the predicted colony radius exceeded the risk threshold. Therefore, β=120 was assigned to enhance the influence of fungal growth risk near the growth warning boundary and to reflect the growth-control-prioritized objective of food storage regulation. For the quality deterioration term Q(t), a scaling coefficient of 400 had already been introduced to balance the different numerical magnitudes of temperature and water activity deviations; thus, η=15 was used to ensure that quality preservation exerted a meaningful but secondary influence relative to fungal growth control. The switching cost S(t) penalized abrupt changes between adjacent regulation modes to improve control smoothness and engineering feasibility; therefore, λ=5 was selected as a moderate penalty to discourage excessive switching while still allowing timely adjustments when fungal growth risk increased. The risk exponential growth coefficient was set to $c_{\mathrm{Risk}}=8$, and the risk threshold was set to $y_{\mathrm{safe}}=1.5$. Previous research [56] had shown that when the diameter of a mold colony reached approximately 3 mm, its contamination status could be visually identified. Therefore, this study used the state corresponding to a colony radius of 1.5 mm as the visually detectable fungal contamination threshold to characterize the entry of food into a state of visually detectable fungal contamination. The specific values of the above parameters could be adjusted according to different food types and actual circumstances.

Because the physiological state variable *q* in the classical Baranyi–Roberts model could reflect the degree of adaptation and activity of microorganisms in the current environment, this study combined the colony radius *y* and the physiological state of the cells, *q*, to construct two representative initial conditions, namely a low fungal growth risk condition and a high fungal growth risk condition, to analyze the response characteristics of the regulation strategy under different risk states. The low risk condition was set to $y_{0}=0.1$ and $q_{0}=0.05$, whereas the high-risk condition was set to $y_{0}=1.3$ and $q_{0}=2$. The storage time *t* was set to 8 days, with 1 day used as each discrete regulation time point, i.e., Δt=1 and $N_{p}=8$.

**Figure 4:**
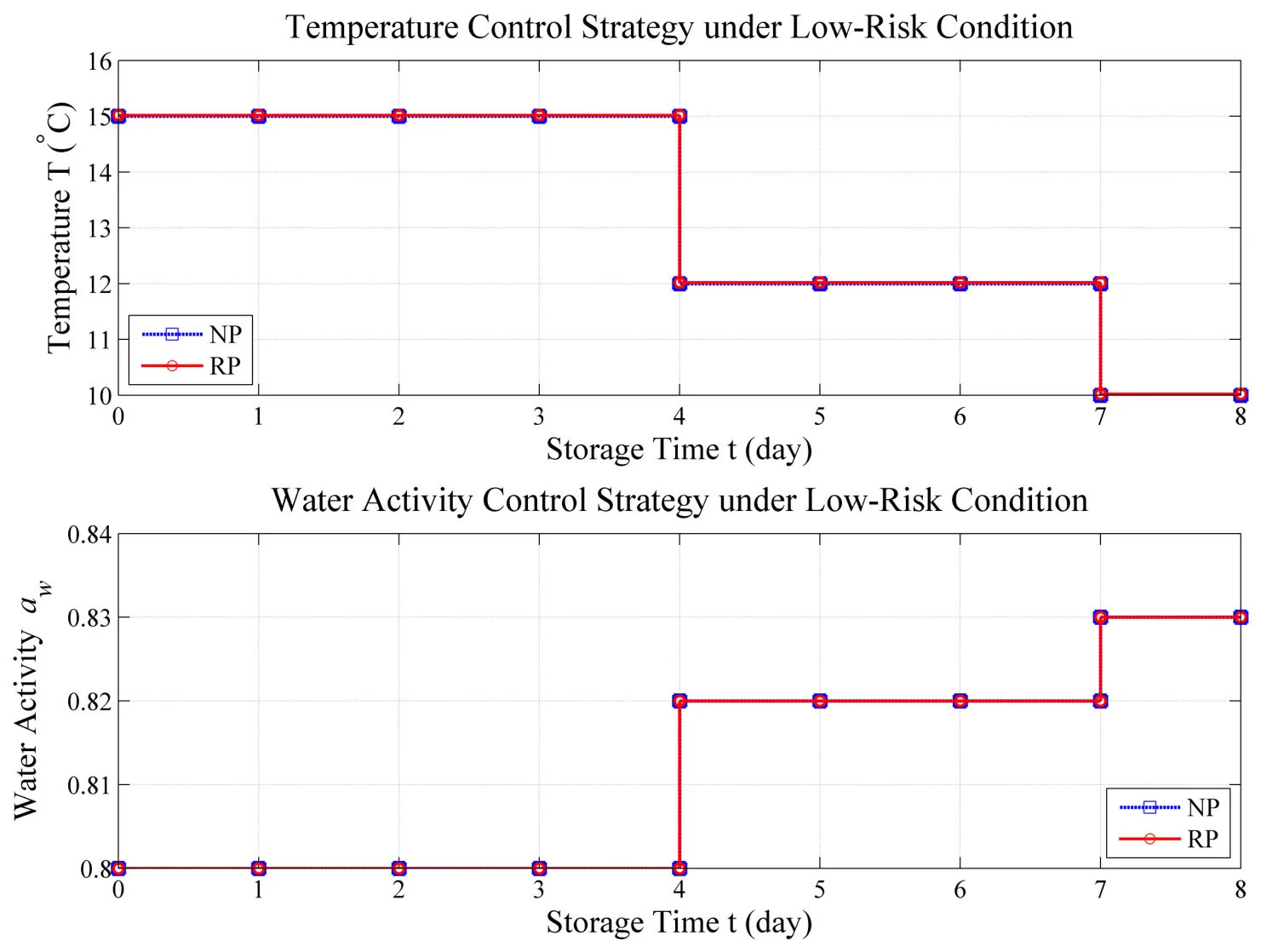
Optimal regulation results for storage strategies under NP and RP in the low-risk operating condition.

For convenience, in this study, nominal prediction was abbreviated as NP and referred to the process of evaluating candidate temperature and water activity sequences using a deterministic fungal growth dynamics model, whereas risk prediction was abbreviated as RP and referred to the prediction process that further incorporated the FRS risk margin. Under the low-risk operating condition, the optimal regulation strategies obtained by NP and RP were shown in Figure 4. Because the system’s nominal prediction trajectory remained far from the growth warning boundary, the trajectory did not exceed the growth warning boundary even when expansion of the risk margin was considered. Therefore, after the risk margin was incorporated, the optimal regulation strategy remained unchanged, and the total cost was 231.0396 in both cases. This result indicated that, under a low-risk scenario, the risk margin did not cause evident excessive conservatism in the system.

**Figure 5:**
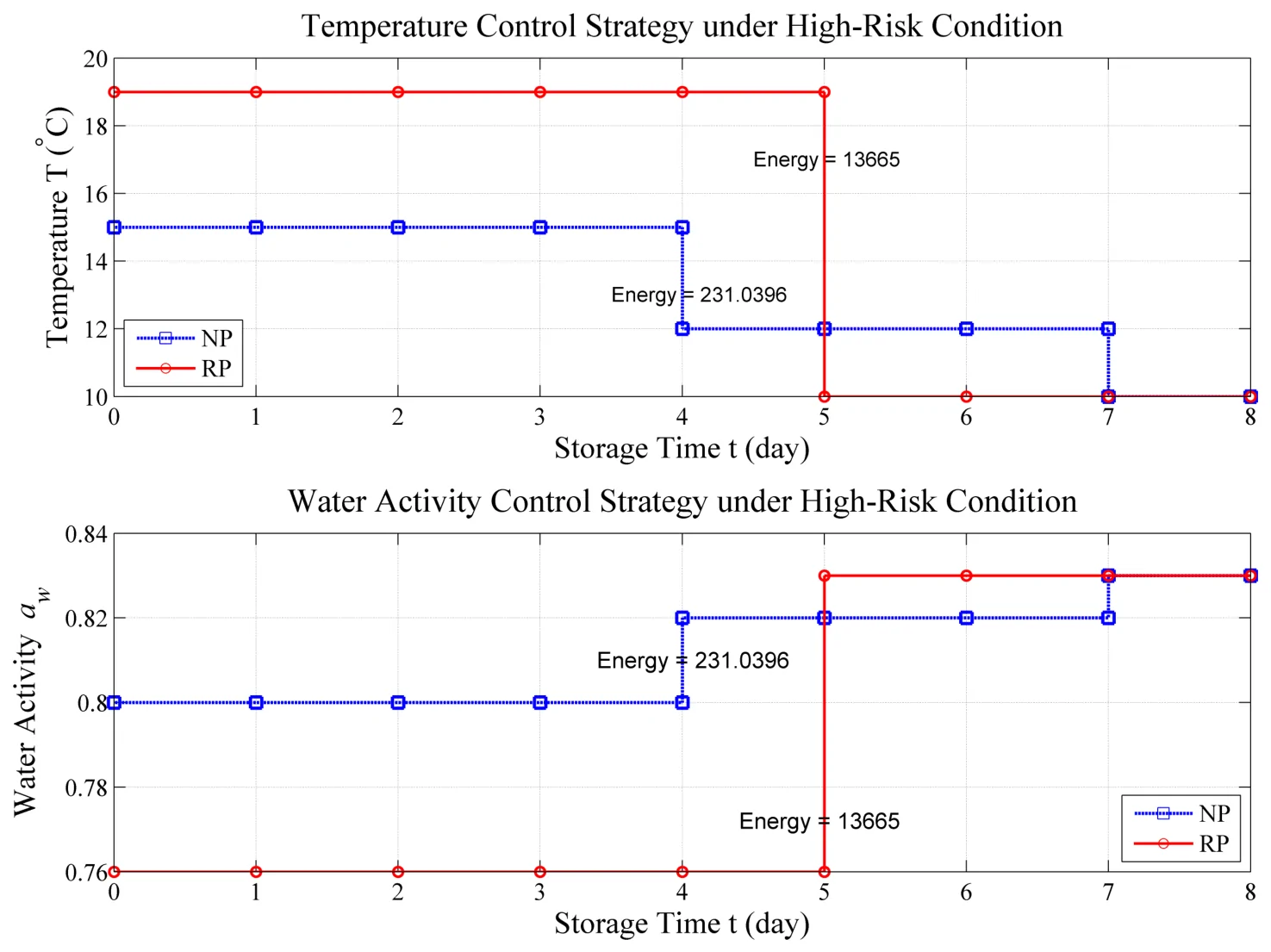
Optimal regulation results for storage strategies under NP and RP in the high-risk operating condition.

**Figure 6:**
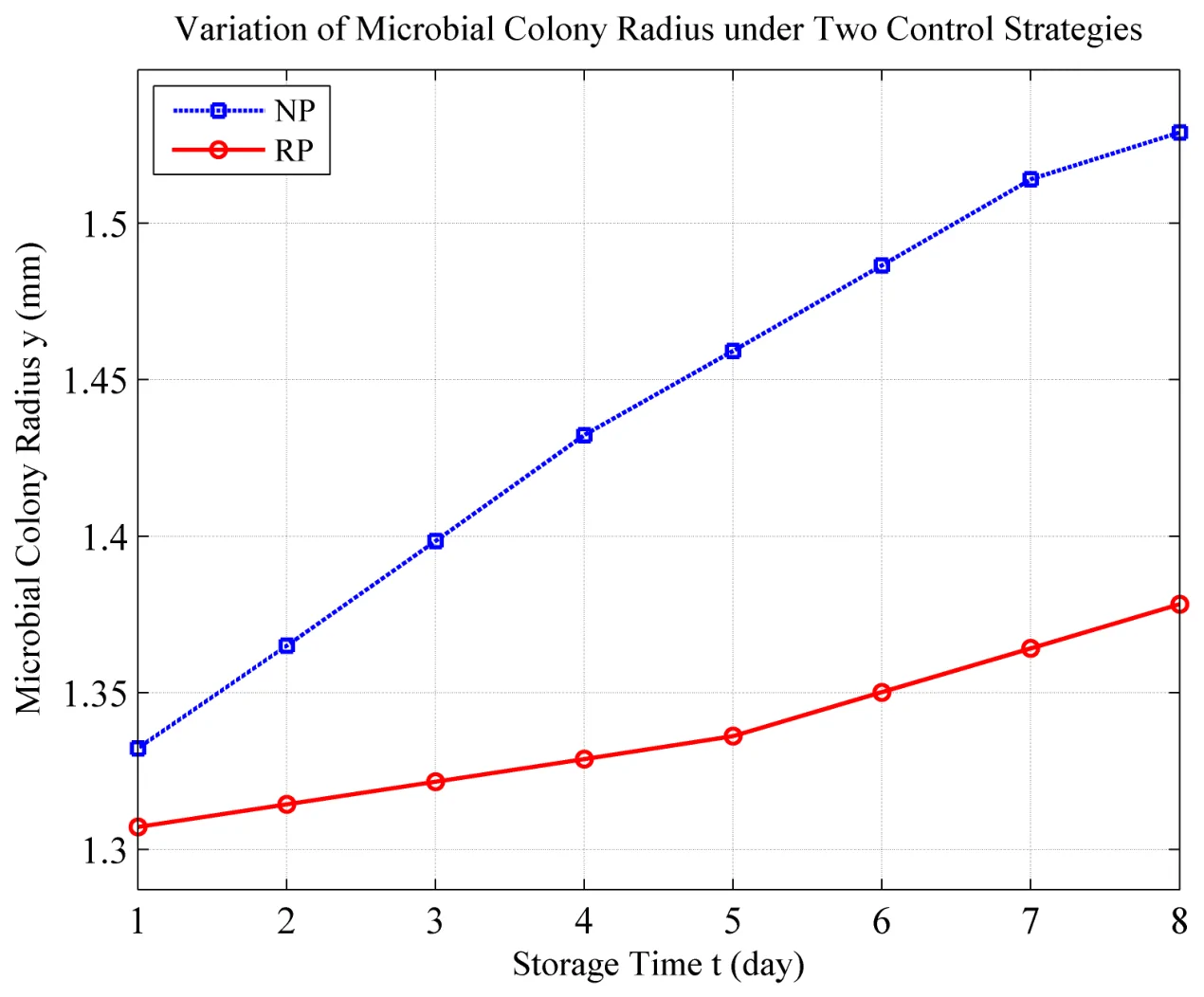
Changes in the fungal colony radius under the optimized storage regulation strategies obtained by NP and RP for the high-risk operating condition.

Under the high-risk operating condition, the optimal regulation strategies corresponding to nominal prediction and risk prediction were shown in Figure 5. Compared with the low-risk operating condition, the optimal environmental regulation strategy of the system changed markedly. The fungal colony radius prediction sequences under the two strategies were further analyzed, as shown in Figure 6. Figure 6 showed that the radius sequence obtained by nominal prediction exceeded the growth warning threshold during the later stage of the prediction horizon. However, after the risk margin vector was introduced, the system could detect the risk propagation trend in advance and proactively switch to a stronger inhibitory mode, thereby slowing the increase in fungal colony radius during the early stage of prediction. Ultimately, the regulation strategy obtained under risk prediction maintained the predicted colony radius below the predefined threshold within the simulation horizon. Although the total cost of risk prediction was substantially higher than that of nominal prediction, further analysis showed that the increase mainly arose from the higher risk cost rather than solely from increased energy consumption associated with environmental regulation. This finding indicated that the risk enhancement mechanism essentially increased the conservatism of the regulation strategy by accepting a higher cost of risk avoidance.

In summary, the risk margin had a limited influence on the regulation strategy under low-risk scenarios. Under high-risk scenariosLax–Friedrichs, the fungal growth risk prediction mechanism prompted the system to adopt stronger inhibitory measures in advance, preventing the predicted colony radius from exceeding the predefined threshold in the simulated scenario and thereby transforming the regulation strategy from traditional risk-responsive regulation to risk-preventive regulation. Meanwhile, this mechanism significantly affected regulation decisions only when the system approached the growth warning boundary, thus enabling a dynamic balance among fungal growth risk control, energy consumption, quality preservation, and regulation smoothness.

## 4. Conclusions

This study proposed a fungal growth risk prediction and optimal regulation method for food storage based on the FRS. Numerical simulation results showed that the constructed risk margin reflected the state expansion caused by the propagation of prior-parameter errors over time. When the predicted state approached the predefined threshold, incorporating the risk margin prompted the system to adopt stronger inhibitory strategies in advance, making the regulation more preventive and conservative. The proposed method provided a numerical framework for fungal growth prediction and storage environment regulation under uncertain conditions. Future studies will combine actual food storage experiments with statistical evaluation to further validate its prediction accuracy and practical applicability.

## Figures and Tables

**Table 1:** Definitions of the main mathematical symbols used in the proposed method.

Symbol	Definition
$x={\left[y,q\right]}^{T}$	System state vector
$u={\left[T,a_{w}\right]}^{T}$	Control input
$X_{0}$	Initial state set
*U*	Control input set
H(x,p)	Hamiltonian function
$p=\frac{\partial \phi (x,t)}{\partial x}$	Hamiltonian costate vector
$X_{t}$	FRS at time *t*
$M\equiv \{m_{1},m_{2},\ldots ,m_{N}\}$	Discrete regulation mode library
$m_{i}={\left[T_{i},a_{w,i}\right]}^{T}$	The *i*-th regulation mode
π	Candidate strategy
*J*	Comprehensive performance evaluation index
E(t),R(t),Q(t),S(t)	Energy consumption cost, fungal growth risk cost, quality deterioration cost, and regulation switching cost, respectively
α,β,η,λ	Weighting coefficients of the corresponding cost terms
$N_{p}$	Number of prediction steps
Δt	Regulation period

**Table 2:** Parameter values for the *A. flavus* growth kinetics model.

Symbol	Value (Unit)
$T_{\min}$	6.33(°C)
$T_{\max}$	42(°C)
$T_{\mathrm{opt}}$	33.47(°C)
$a_{w_{\min}}$	0.74(−)
$a_{w_{\mathrm{opt}}}$	0.99(−)
$\mu_{\mathrm{opt}}$	4.31(mm/d)
$y_{M}$	10(mm)

**Table 3:** Temperature–water activity control mode library for food storage.

Mode Number	Temperature *T* (°C)	Water Activity $a_{w}$
$m_{1}$	10	0.83
$m_{2}$	12	0.82
$m_{3}$	15	0.80
$m_{4}$	17	0.78
$m_{5}$	19	0.76

## Data Availability

The original contributions presented in this study are included in the article. Further inquiries can be directed to the corresponding author.

## Abbreviations

The following abbreviations are used in this manuscript:

FRS	forward reachable set
BRS	backward reachable set
NP	nominal prediction
RP	risk prediction
